# Impact of neutrophil extracellular traps on fluid properties, blood flow and complement activation

**DOI:** 10.3389/fimmu.2022.1078891

**Published:** 2022-12-16

**Authors:** Antonia Burmeister, Sabine Vidal-y-Sy, Xiaobo Liu, Christian Mess, Yuanyuan Wang, Swagata Konwar, Todor Tschongov, Karsten Häffner, Volker Huck, Stefan W. Schneider, Christian Gorzelanny

**Affiliations:** ^1^ Department of Dermatology and Venereology, University Medical Center Hamburg-Eppendorf, Hamburg, Germany; ^2^ Department of Internal Medicine IV, Medical Center, Faculty of Medicine, University of Freiburg, Freiburg, Germany

**Keywords:** complement, coagulation, DNA, immunothrombosis, neutrophil extracellular traps, blood rheology, blood viscosity

## Abstract

**Introduction:**

The intravascular formation of neutrophil extracellular traps (NETs) is a trigger for coagulation and blood vessel occlusion. NETs are released from neutrophils as a response to strong inflammatory signals in the course of different diseases such as COVID-19, cancer or antiphospholipid syndrome. NETs are composed of large, chromosomal DNA fibers decorated with a variety of proteins such as histones. Previous research suggested a close mechanistic crosstalk between NETs and the coagulation system involving the coagulation factor XII (FXII), von Willebrand factor (VWF) and tissue factor. However, the direct impact of NET-related DNA fibers on blood flow and blood aggregation independent of the coagulation cascade has remained elusive.

**Methods:**

In the present study, we used different microfluidic setups in combination with fluorescence microscopy to investigate the influence of neutrophil-derived extracellular DNA fibers on blood rheology, intravascular occlusion and activation of the complement system.

**Results:**

We found that extended DNA fiber networks decelerate blood flow and promote intravascular occlusion of blood vessels independent of the plasmatic coagulation. Associated with the DNA dependent occlusion of the flow channel was the strong activation of the complement system characterized by the production of complement component 5a (C5a). Vice versa, we detected that the local activation of the complement system at the vascular wall was a trigger for NET release.

**Discussion:**

In conclusion, we found that DNA fibers as the principal component of NETs are sufficient to induce blood aggregation even in the absence of the coagulation system. Moreover, we discovered that complement activation at the endothelial surface promoted NET formation. Our data envisions DNA degradation and complement inhibition as potential therapeutic strategies in NET-induced coagulopathies.

## Introduction

Intravascular blood clotting occurs in a large variety of diseases such as antiphospholipid syndrome, COVID-19 or vasculitis (1–4). Blood vessel occlusion is a severe complication and mostly associated with pain, tissue damage and organ failure. The causes of hypercoagulation and formation of intravascular clots are diverse with a large variety between the different disease entities and inter-individual differences. Although exact molecular triggers promoting hypercoagulation are often elusive, therapy with anticoagulants such as factor Xa (FXa) inhibitors or low molecular weight heparins can ameliorate diseases symptoms.

The coagulation system, a cascade of consecutive proteases is highly complex and strictly regulated at different steps by a plethora of factors. Conventionally, the coagulation system is divided into the intrinsic and extrinsic pathway. The starting point of the intrinsic pathway is FXII, which is converted to its activated form FXIIa upon contact to collagen. The extrinsic pathway is initiated by tissue factor, which is e.g., expressed by activated endothelial or immune cells. The intrinsic and extrinsic coagulation pathway converge into the same final part of the plasmatic coagulation also referred to as the common pathway. The common pathway culminates in the formation of thrombin, which catalyzes the conversion of fibrinogen into fibrin. Fibrin, the final product of the coagulation cascade forms a polymer network trapping circulating blood cells such as platelets to stop blood flow.

Evolutionary tightly connected to the coagulation cascade is the complement system, an ancient but powerful part of the innate immunity (5). In analogy to the coagulation system, the action of the complement system bases on a variety of interacting proteins such as the complement component 3 (C3) or C5. Proteolytic cleavage of C5 by the C5 convertase results in the formation of C5a and C5b. While C5a is a strong chemotactic molecule for neutrophils, C5b is part of the membrane attack complex (MAC) building a cell lytic membrane pore into complement attacked cells. Traditionally, the complement system is split into three complement pathways the classical pathway, the lectin pathway and the alternative pathway. To highlight the close connection to the coagulation system some researchers refer to the extrinsic complement pathway describing the cleavage of C5 into C5a and C5b by thrombin (6).

Mechanistically connected to coagulation and complement activation is the formation of C5a and thus the recruitment and activation of neutrophils. Interestingly, strong neutrophil activation e.g., through the thrombin-sensitive protease activated receptor 2 (PAR-2) is known to promote the release of decondensed chromosomal DNA also known as neutrophil extracellular traps (NETs) (7). In the context of infections, NETs were shown to trap invading microorganisms (8), to activate the complement system (9) and to be procoagulatory (10). Procoagulatory properties of NETs have been explained by different pathways. For example, NETs were considered to initiate the coagulation cascade by promoting activation of FXII (11) or through the exposure of tissue factor (12, 13). We have previously shown that NETs can interact with procoagulant von Willebrand factor (VWF) (14). Similar to fibrin, vWF can form polymeric networks within the lumen of thrombotic blood vessels leading to the entrapment of platelets (15), erythrocytes (16) and immune cells (17). VWF exhibits a dynamic shear stress dependent biological activity (18). Critical shear stress results in the elongation of vWF, which is accompanied with the exposure of the platelet binding region within the A1 domain. Although this shear sensing confers unique biological properties to vWF, the conformational change of a polymer under flow is a common phenomenon. In dependence of the molecular properties of the polymer, such fluids change their viscosity as a function of the applied shear rate. This behavior is also known as non-Newtonian.

In the past, rheological properties of polymer (e.g., DNA) solutions have been extensively investigated. At high DNA concentrations, DNA chain entanglement is associated with supramolecular organization. This produces a non-Newtonian fluid behavior characterized by a decreasing viscosity of the solution at increasing shear rates (19, 20). This shear thinning behavior of DNA solutions has been studied with purified DNA molecules. Because DNA is the major component of NETs, it is likely that they contribute to coagulation by affecting the rheology of the blood flow even in the absence of the extrinsic or intrinsic coagulation cascade.

In the present study, we investigated the impact of NETs on blood flow properties at different shear stress conditions. NET related change of the blood rheology is further connected to blood vessel occlusion and the activation of the complement system. Moreover, we analyzed the impact of complement activation on NET release.

## Material and methods

### Cell culture

Human umbilical vein endothelial cells (HUVEC) were freshly isolated from umbilical cords as previously reported (21). Briefly, umbilical cords were separated from the placenta, cords were cleaned with 1ml of PBS per cm, infused with Collagenase 0.1% (Clostridium histolyticum, Sigma-Aldrich, USA), pinched off at each end and incubated in a container of PBS placed in a 37° water bath for 10 minutes. Detached HUVECs were then collected and centrifuged at 284 g for 5 minutes. HUVECs were cultured at 37°C and 5% CO_2_ in a humidified atmosphere in culture medium (one-third endothelial cell growth medium-2 (Lonza, Switzerland); two-thirds M199 medium (Gibco Life technology corporation NY, USA) supplemented with 10% fetal bovine serum (Sigma-Aldrich), 1% penicillin and streptomycin. For microfluidic experiments, HUVECs (350.000 cells per slide) were seeded into microfluidic channels (μ-slide I Luers^0.2mm^ Ibidi, Gräfelfing, Germany). Prior to the seeding, slides were coated with 0.5% gelatin.

### Microfluidic experiments with whole blood

HUVECs were perfused with hirudinated whole blood at indicated shear rates as previously reported (22). Prior to the perfusion, blood was either left untreated or supplemented with 15 nM phorbol-myristat-acetate (PMA, Sigma-Aldrich), 100 U/ml DNase I I (Sigma-Aldrich), 100 µM protein arginine deiminase (PAD) 4 inhibitor Cl-Amidine (Cl-A, Sigma-Aldrich), 50 mg/ml complement activating heat aggregated gammaglobulins (c-Ig) dissolved in PBS or 2 mg/ml anti-glycoprotein (GP) IIb/IIIa antibodies (Abciximab, Lilly Deutschland GmbH, Germany). The applied dose of 15 nM PMA was evaluated to be the minimal dose required to induce NETosis in whole blood. DNA was stained with DAPI (Roche, Switzerland) or EvaGreen (Jena Bioscience, Germany). Where indicated, HUVECs were pre-labeled with an anti CD31 antibody (Rat IgG2a, Biolegend, USA) to induce complement activation (23). Microfluidic slides were mounted to the stage of an inverted fluorescence microscope (Observer Z.1, Zeiss, Germany) and incubated at 37°C during the whole experiment to image the release of NETs. After 4h of continuous unidirectional perfusion, 4 ml blood was immediately collected and centrifuged at 800 g for 5 minutes. Isolated plasma was aliquoted and stored at -20°C to avoid further complement activation. Channels were fixated with paraformaldehyde 4% for 10 minutes and subsequently washed with 4-(2-hydroxyethyl)-1-piperazineethanesulfonic acid (HEPES) buffered Ringer’s solution. Slides were stained with CD15 (Abcam, UK), C3d (Abcam), CD31 (Agilent, USA), MAC (Hycult biotech, USA), tissue factor (GeneTex, USA) and thrombospondin-1 (Invitrogen, USA) directed antibodies as previously reported (23) and imaged by fluorescence microscopy (Observer Z.1, Zeiss). Occlusion areas and numbers of neutrophils were analyzed by ImageJ version 1.53r (24).

### ELISA

Complement activation was quantified by C5a ELISA (Human Complement Component C5a DuoSet ELISA, DY2037 R&D Systems, Inc., USA). The ELISA was performed according to the manufactures protocol with modification. Briefly, to prevent further complement activation during the incubation period we added 30 µl ice cold 20 mM EDTA to the samples. Absorbance was measured using a microplate spectrophotometer (Biotek PowerWave XS, USA) at a wavelength of 450nm. Citrullinated Histone 3 was measured using a sandwich ELISA Kit as suggested by the manufacturer (Clone 11D3, Cayman Chemical, USA). Cell death detection ELISA PLUS (Roche, Switzerland) was used to detect histones H1, H2a, H2b, H3 and H4, single and double stranded DNA. A chromogenic assay (CoaChrom^®^ Diagnostica GmbH, Austria) was used to measure factor XIIa-like activity. Cell death detection ELISA PLUS and XIIa activity assay were performed according to the manufacturer’s instructions.

### Microfluidic experiments with isolated NETs and microparticle velocimetry

To investigate the impact of isolated NETs on blood flow we used two different approaches. First, we coated microfluidic channels with a height of 200 µm (μ-slide I Luers^0.2mm^ Ibidi) with anti-CD15 antibodies to immobilize human neutrophils isolated from peripheral blood as previously reported (23). Prior to the perfusion, neutrophils were stimulated with 1000 µM PMA for 2h at 37°C. DAPI and EvaGreen (Jena Bioscience, Germany) were added to visualize DNA. Netting neutrophils were perfused at indicated shear rates with a HEPES buffered Ringer’s solution containing 0.02% w/v fluorescent polystyrene microparticles (size 0.84 µm, purple, Kisker Biotech, Germany). Second, we separated NETs from 7×10^6^ neutrophils using a mild DNA extraction procedure (QuickExtract, Lucigen, USA) producing DNA with a high protein content. In control experiments, NETs were further purified (DNAzol, Thermofischer, USA) to remove protein contaminations. NETs or purified DNA were diluted in HEPES-buffered Ringer’s solution containing 0.02% w/v fluorescent polystyrene microparticles (size 0.84 µm, purple, Kisker Biotech, Germany) and transferred to microfluidic channels with a height of 70 µm (BioFlux 48 well plate 0-20 Pa, Fluxion Biosciences, USA). Solutions were perfused at indicated shear rates and as previously reported (25).

Trajectories of the flowing particles were recorded with a fluorescence microscope (Observer Z.1, Zeiss) at different focal planes ranging from the bottom to the top of the channel. We developed an OpenCV based image processing pipeline to automatically measure the velocimetry of the flowing particles in each image frame (26). This pipeline applies Gaussian smoothing in a first step for image noise reduction and afterwards enhances tubular like structures *via* Frangi filtering (27). An automatic thresholding algorithm (Otsu’s method) is utilized to identify the most dominant particle trajectories which are morphometrically filtered for final measurements (28).

In relation to their height position *z* within the fluidic channel, velocities *ν* of the particles were fitted to the analytical solution of the Navier-Stokes solution for rectangular channels as previously reported (29, 30),


$$v\left(z\right)=-\frac{4h^{2}}{\eta \pi^{3}}\left(\frac{dp}{dx}\right)\sum_{n=0}^{\infty }\frac{1}{{\left(2n+1\right)}^{3}}\left[1-\frac{\cosh\left(\left(2n+1\right)\pi \frac{z}{h}\right)}{\cosh\left(\left(2n+1\right)\pi \frac{w}{2h}\right)}\right]$$

where *dp/dx* is the pressure drop within the channel and η the viscosity of the solution flowing through the channel with height *h* and width *w*.

### Statistical analysis

Data are presented as mean ± SD. Data were analyzed with GraphPad Prism version 9.4.1 (GraphPad Software) and OriginPro version 2022b (OriginLab Corporation). Significance was tested using two-tailed Student’s *t*-test, one-way ANOVA with Tukey or with Dunnett’s T3 *post hoc* test or Fisher *z*-transformation. Differences were considered statistically significant at *p<0.05 and **p<0.01, ***p<0.0005, ****p<0.0001.

## Results

### Presence of neutrophil derived DNA correlates with blood aggregation

**Figure 1:**
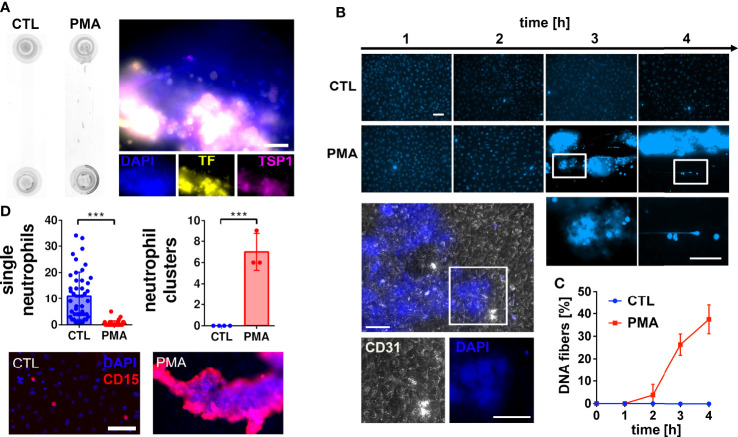
Neutrophil extracellular trap (NET) formation in hirudinated whole blood. **(A)** HUVECs were seeded into microfluidic channels and perfused with hirudinated blood at a shear stress of 0.5 Pa for 4 h. NETosis was induced by 15 nM PMA. Macroscopic blood aggregations were formed in PMA-treated slides but not in corresponding controls (CTL). Representative immunofluorescence staining of PMA treated slides indicates that aggregates contain DNA (DAPI), tissue factor (TF) and thrombospondin-1 (TSP1). Scale bar = 50 µm **(B)** Representative fluorescence microscopy images of DAPI stained slides during the experiment. CD31 staining (white) shows that the endothelium underneath the DNA-rich aggregates remained intact. Nuclei of endothelial cells are not visible because the brightness and contrast settings of the images were adjusted to the strong fluorescence signal emitted by the DNA aggregates. White boxes indicate magnified areas. Scale bar = 50 µm. **(C)** Quantitative analysis of the time series experiment shows that the number of DNA fibers increased time-dependently. **(D)** Fluorescence microscopic images of neutrophils after the flow experiment. Quantifications of images are presented in bar diagrams indicating the number of single neutrophils and neutrophil clusters in control slides in comparison to PMA-treated slides. Scatter plots indicate the number of analyzed fields of view of three independent experiments (single neutrophils) or the number of neutrophil clusters per slide of three independent experiments (neutrophil clusters). Representative fluorescence images of single neutrophils and neutrophil clusters are shown. CD15 was used as neutrophil surface marker (red). Scale bar = 100 µm. ***P<0.001, Student’s *t*-test.

To investigate the impact of neutrophil extracellular DNA on blood flow and blood clotting, we stimulated neutrophils with PMA in human hirudinated whole blood. PMA is a well-known neutrophil activator inducing the release of NETs (8). To mimic flow conditions in postcapillary venules (31) we applied a shear stress of 0.5 Pa. **Figure 1A** shows that PMA treatment promoted the formation of macroscopic aggregates within the flow channel. Immunofluorescence analysis indicates that aggregates contain DNA, tissue factor (TF) and thrombospondin-1 (TSP1). Because we performed our experiments in hirudinated blood, TF-triggered thrombin formation was considered to be prevented. Channel occlusion was a time-dependent effect. Therefore, we quantified the number of DNA fibers released by neutrophils over the course of continuous blood perfusion by fluorescence microscopy (**Figures 1B, C**). Our data demonstrates that macroscopic aggregates of neutrophils and extracellular DNA were already formed 3 hours after stimulation with PMA. Of note, underneath these clot-like structures, the endothelium remained intact as indicated by CD31 staining (**Figure 1B**). **Figure 1D** shows that in control channels, neutrophils were evenly distributed and appear as single cells. In contrast, neutrophils form large clusters upon PMA stimulation.

**Figure 2:**
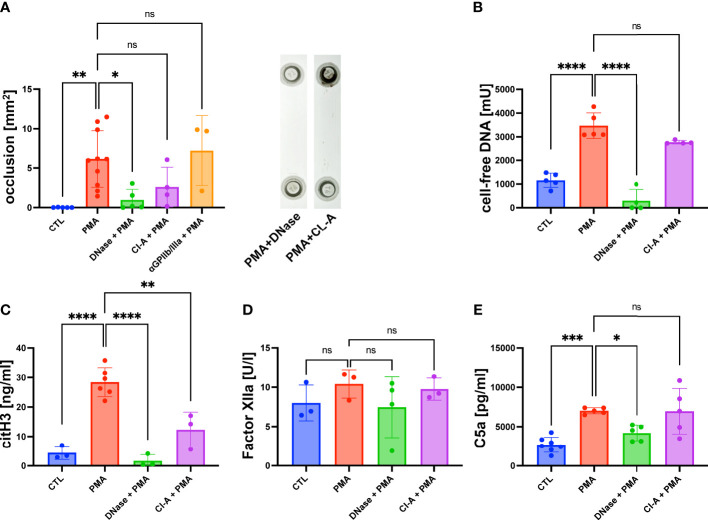
PMA induced channel occlusion and effects of DNA degradation and NETosis inhibition in hirudinated whole blood. **(A)** Quantitative analysis of the channel occlusion is shown as bar diagram. To prove the impact of extracellular DNA and NETosis on occlusion, DNase I or Cl-A was added. Representative photographs of fluidic slides, after treatment with DNase I or CL-A are shown. **(B)** Plasmatic cell-free DNA was increased in PMA-treated slides in comparison to CTL slides. Amount of cell-free DNA was reduced by DNase I and Cl-A treatment. **(C)** CitH3 was increased in PMA-treated slides in comparison to CTL slides. Less plasmatic citH3 was measured upon DNase I or Cl-A treatment. **(D)** Plasmatic levels of factor XIIa were neither affected by PMA stimulation nor DNase I or Cl-A treatment. **(E)** C5a was significantly increased in PMA-treated slides in comparison to CTL slides. PMA-induced C5a levels were significantly decreased upon DNase I treatment. Data are shown as mean ± SD, scatter plots indicate the number of independent experiments, *p<0.05, p**<0.01, ***p<0.0005, ****p<0.0001, ns = not significant, one-way ANOVA with Tukey *post hoc* test.

As shown in **Figure 1A**, the accumulation of DNA within the lumen of the flow channel correlated with the formation of macroscopic clot-like structures. In PMA-stimulated slides, we measured an occlusion area of 5.2 ± 1.79 mm^2^, whereas no occlusions were detectable in control slides (**Figure 2A**). To verify that cell-free DNA is fundamental for aggregate formation, we investigated the effect of DNase I I and the NETosis inhibitor Cl-A (**Figure 2A**). DNase I I is a mammalian serum endonuclease that hydrolyzes single and double stranded extracellular DNA. Cl-A inhibits PAD 4, which is an enzyme involved in the process of NETosis as it citrullinates histones and by that facilitates chromatin decondensation (32). **Figure 2A** shows a significantly reduced occlusion area after administration of DNase I I compared to the PMA group. There was also a clear trend towards reduced DNA aggregation after PAD4 inhibition, but CL-A was less efficient than the DNase I treatment. In control experiments, we added a monoclonal antibody directed against GPIIb/IIIa to the perfusing blood. GPIIb/IIIa is exposed by activated platelets and involved in platelet aggregation. As shown in **Figure 2A**, the sizes of clot-like structures were not affected by GPIIb/IIIa neutralization.

After the flow experiment, we collected the blood and prepared plasma samples for further analysis using ELISA. The levels of cell-free DNA were significantly increased after PMA stimulation compared to the control samples (**Figure 2B**). DNase I treatment antagonized the effect of PMA significantly. PAD4 inhibition by CL-A after PMA stimulation was less efficient, but a clear trend of reduced cell-free DNA was detectable. In line with the data shown in **Figure 2A**, DNase I was more efficient than PAD4 inhibition (**Figure 2B**). To confirm that cell-free DNA was derived from netting neutrophils, we measured the plasma levels of citrullinated Histone 3 (citH3), which is a marker for NETosis. **Figure 2C** shows that PMA-stimulated blood contained significantly more citH3 than blood obtained from control experiments. PMA-induced CitH3 levels were decreased by DNase I and Cl-A treatment (**Figure 2C**). Previous studies suggested that the negatively charged surface of NETs can serve as an initiation platform for the intrinsic pathway of the coagulation cascade (33). In a chromogenic assay we determined the concentration of factor XIIa to quantify intrinsic pathway activation in our flow experiments. **Figure 2D** shows that the levels of FXIIa were not increased in slides with high levels of cell-free DNA suggesting that the intrinsic coagulation cascade was not triggered. In line with that, also DNase I and CL-A treatment had no impact on FXIIa concentrations. As complement activation on the surface of released NETs has been described in literature (34) we measured the amount of human complement component C5a in PMA-stimulated plasma samples and control plasma. **Figure 2E** shows significantly increased levels of C5a in PMA plasma compared to the control group, suggesting that NETosis was associated with complement activation. Interestingly, DNase I I treatment resulted in a significantly diminished concentration of C5a suggesting a molecular connection between NET release and the complement system (**Figure 2E**).

### Activation of the complement system promoted NET release and channel occlusion

**Figure 3:**
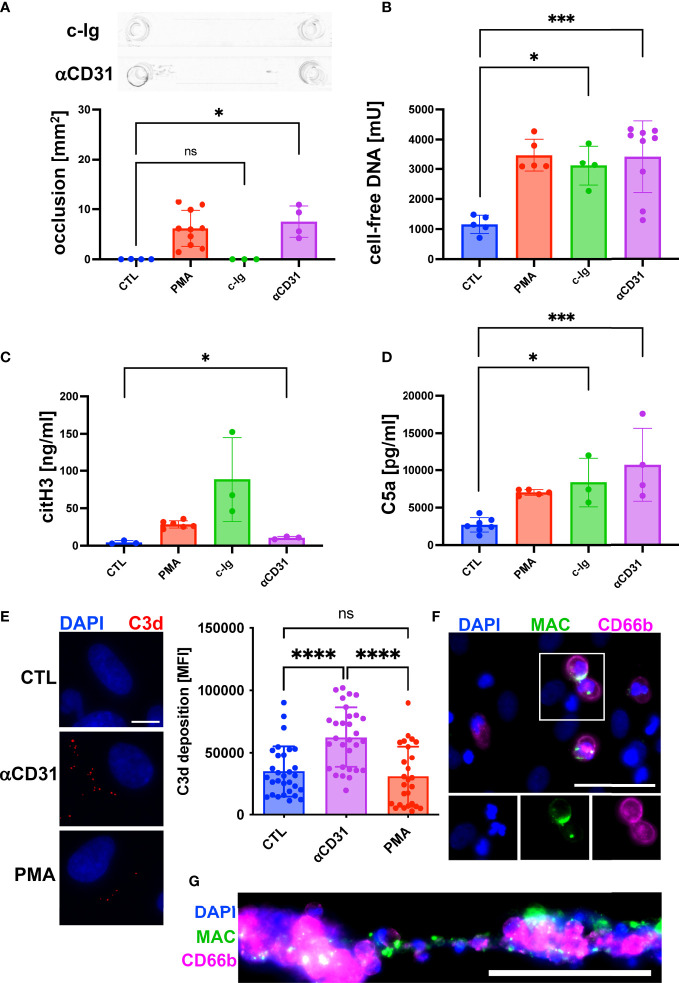
Activation of complement system promoted NETosis and channel occlusion in hirudinated whole blood. To induce complement activation in the fluid phase, complexes of heat aggregated immunoglobulins (c-Ig) were added to the perfusing blood. To induce complement activation at the endothelium, CD31 directed antibodies (αCD31) were deposited on the surface of HUVECs prior to the perfusion with whole blood. **(A)** No macroscopic channel occlusions were formed in c-Ig-treated slides, whereas channels occluded upon αCD31 treatment. **(B)** Plasmatic cell-free DNA was significantly increased in c-Ig- and αCD31-treated slides in comparison to the CTL group. **(C)** Concentration of plasmatic citH3 was significantly increased in αCD31-treated slides in comparison to the CTL group. Although c-Ig treatment increased the plasmatic concentration of citH3, the effect was statistically not significant. **(D)** Detection of C5a in plasma samples. In comparison to the CTL slide, C5a was significantly increased in c-Ig- and αCD31-treated slides. **(E)** Vascular deposition of C3d (red) was analyzed by immunofluorescence staining of flow slides after the perfusion experiment. Representative fluorescence microscopic images are shown. Scale bar = 10 µm. Quantitative analysis of microscopy images is indicated as bar diagram. **(F)** MAC staining (green) of single neutrophils attached to the endothelium and **(G)** neutrophils/NET conglomerates. CD66b (magenta) was used as a neutrophil marker. Scale bars = 50 µm. For reference, data of PMA-treated and CTL slides, which were already shown in Figure 2, are depicted again **(A-D)**. Data are shown as mean ± SD, scatter plots indicate the number of independent experiments **(A-D)** or the mean fluorescence intensity (MFI) of the C3d staining per field of view of three independent experiments **(E)**, p*<0.05, p***<0.0005, p****<0.0001, ns = not significant, one-way ANOVA with Tukey **(A-E)** or Dunnett’s T3 **(C)** *post hoc* test.

Our results of an increased C5a concentration upon formation of clot-like structures indicated that the complement system was activated. To further understand the role of the complement system during DNA-mediated blood aggregation, we repeated our flow experiments. Instead of PMA, we added either complexes of immunoglobulins (c-Ig) to the perfusing blood to induce complement activation in the fluid phase or we pre-incubated the endothelium with an anti-CD31 directed rat IgG2a antibody (αCD31) to induce complement activation at the endothelial surface. The results shown in **Figure 3A** indicate that c-Ig was not able to induce channel occlusions. In contrast, we measured occlusions in channels pre-incubated with αCD31 (**Figure 3A**). Interestingly, c-Ig treatment and pre-incubation of the endothelium with αCD31 increased the levels of cell-free DNA and C5a significantly (**Figures 3B, D**). Levels of citH3 were significantly increased in αCD31 pre-treated slides and a trend towards higher citH3 levels was found upon c-Ig treatment (**Figure 3C**). To confirm endothelial cell-associated complement activation upon αCD31 pre-incubation, we measured C3d deposition at the endothelium after the flow experiment by immunofluorescence analysis. C3d is a downstream product of C3b, which is covalently attached to complement activated surfaces (23). **Figure 3E** shows increased levels of C3d on endothelial cells that had been pre-incubated with αCD31 prior to the flow experiment suggesting complement activation at the endothelium. The end point of the complement cascade is the MAC, which forms a lytic pore in the membrane of complement attacked cells. Most mammalian cells are protected against MAC deposition due to the expression of CD59. However, we recently found that neutrophils, which are in close physical proximity to complement attacked endothelial cells, acquire MAC from the endothelium and undergo NETosis (23). In line with this, we found MAC deposition on neutrophils and NET conglomerates (**Figures 3F, G**).

Taken together, our data indicate that the complement is not only triggered during NETosis but that the activation of the complement system at the blood vessel wall can further fuel NET release.

### NETs decelerating liquid flow by increasing fluid viscosity

**Figure 4:**
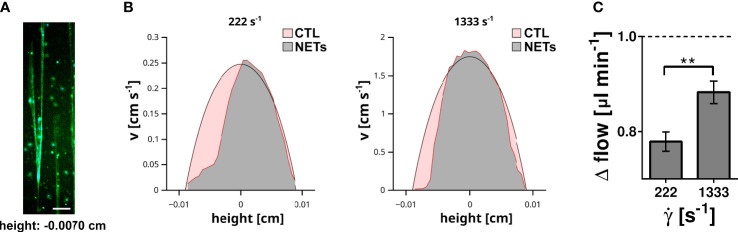
Impact of surface immobilized NETs on fluid flow. **(A)** NETs immobilized at the bottom (height = -0.007 cm) of the flow channel. DNA was stained with DAPI (blue) and EvaGreen (green). Scale bar = 100 µm. **(B)** The velocity (v) of microparticles within the flow channel was measured by microparticle velocimetry. Flow velocity profile recorded at a shear rate of 222 s^-1^ and 1333 s^-1^ are shown. The profile measured in control channels (red area) were superimposed with the profile measured in NET containing channels (grey area). **(C)** Impact of the shear rate (˙γ) on the total fluid flow. Shown is the change of the total flow in comparison to the corresponding flow within the control channels. The flow in the control channel is indicated by the dashed line. **p< 0.01, Fisher *z*-transformation.

NETs and DNA are macromolecules with an extraordinary high molecular weight converting the flowing solution into a non-Newtonian fluid with a shear stress dependent viscosity. Previous data indicate that flow-directed orientation of DNA chains correlate with a decrease of the viscosity (35). To assess whether NETs affect the fluid properties of a flowing solution we performed a series of microparticle velocity experiments. We immobilized neutrophils at the bottom of microfluidic channels and induced NETosis by PMA (**Figure 4A**). NETs were perfused with buffer containing microparticles (diameter = 0.89 µm). Plain polystyrene particles were used to avoid direct molecular interactions between cell-free DNA and the flowing particles. Particle velocities were measured at different height positions within the microfluidic channel by fluorescence microscopy. In control channels, we obtained a typical parabolic flow profile in which the highest particle velocity was in the center of the channel (height = 0 cm), while flow velocity approached zero towards the bottom (height = -0.009 cm) or top of the channel (height = 0.009 cm). In channels containing NETs, the velocity profile deviated from the parabolic shape and the impact of NETs was shear stress dependent (**Figure 4B**). At a shear rate of 222 s^-1^, flow of the particles was almost completely blocked in close proximity to the NETs (channel height -0.007 cm). At a higher flow velocity (shear rate 1333 s^-1^), the presence of NETs was still able to decelerate the flowing particles (**Figure 4B**). However, quantification of the change of the total flow (Δ flow) showed that the impact of NETs was less prominent at higher shear rates (**Figure 4C**). Taken together these experiments indicate that NETs have a strong ability to hinder blood flow and that the impact of NETs was shear stress dependent.

**Figure 5:**
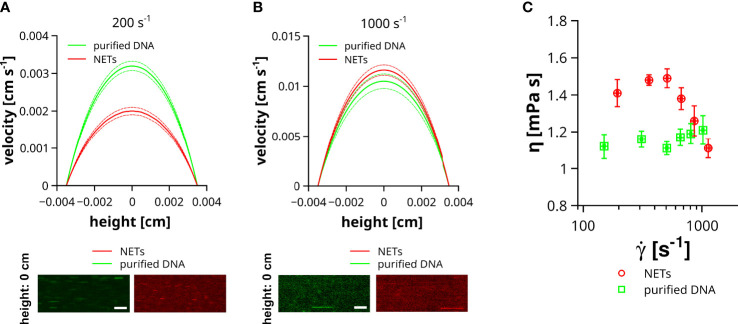
Impact of NETs on fluid viscosity. Velocity (v) of microparticles was measured in channels containing NETs or purified DNA. Flow experiments were performed at different shear rates ranging from 150 to 1129 s^-1^. **(A)** Representative velocity profiles at low shear rates. Image insets show the trajectories of microparticles in the center of the channel (height = 0 cm) either filled with fluid containing purified DNA (green) or NETs (red). Dashed lines indicate the standard deviation. Exposure time = 10 ms, Scale bar = 50 µm. **(B)** Representative velocity profiles at high shear rates. Image insets show the trajectories of microparticles in the center of the channel (height = 0 cm) either filled with fluid containing purified DNA (green) or NETs (red). Dashed lines indicate the standard deviation. Exposure time = 1 ms, Scale bar = 50 µm. **(C)** Shown is the viscosity (η) of the flowing fluid as a function of the applied shear rate (γ˙).

Previous studies indicated the impact of DNA on liquid properties and the shear thinning behavior was connected to a conformational change of the DNA (20). DNA is a very rigid molecule and the intermolecular forces are comparably weak, which is also in line with a stretching of a coiled DNA already at low shear rates (36) and thus reduced impact on the flow of the liquid at high flow velocities. In contrast to purified DNA, NETs are decorated with a plethora of proteins and other neutrophil-derived molecules, which can contribute to stronger intermolecular interactions, crosslinking and entanglement of DNA strands. To which extent the structural difference between DNA and NETs can affect the properties of a flowing liquid was addressed in the following experiment. NETs were isolated from PMA-stimulated neutrophils and either directly used for flow experiments or further purified to remove DNA-bound molecules. In our experiments, we compared the impact of NETs and purified DNA on liquid flow at different shear rates, ranging from 150 ± 3.2 to 1129 ± 18.7 s^-1^. Of note, NETs and DNA were not surface immobilized to prevent changes of the shear by DNA-mediated occlusions of the flow channel. **Figure 5A** shows representative velocity profiles of microparticles at low shear rates (200 s^-1^). Maximal velocity of microparticles (channel height = 0 cm) in liquid containing NETs was 1.6 times slower than in slides containing purified DNA. At higher shear rates (1000 s^-1^) this difference in microparticle velocity was absent (**Figure 5B**). To enable a more comprehensive comparison between liquids containing purified DNA or NETs, we fitted the velocity profiles applying the previously reported analytical solution of the Navier-Stokes equation for rectangular flow channels (29). Using this approach, we were able to link the dynamic viscosity of the fluid (η) to the applied shear rate (γ˙). As shown in **Figure 5C**, the viscosity of the liquid containing purified DNA was, in the here tested range of flow velocities, independent of the applied shear rate. In contrast to the solution with purified DNA, liquids with NETs had a significantly increased viscosity at shear rates below 500 s^-1^. Above 500 s^-1^ viscosity steeply decreases with increasing shear and approaches the viscosity level of solutions with purified DNA at around 1000 s^-1^.

## Discussion

Coagulation, platelet activation and NETosis are closely related molecular partners that were shown to trigger thrombosis in a variety of different diseases (37, 38). Previous studies suggest that NETs and in particular NET-bound histones can promote platelet binding and activation (33, 39). Histones were shown to activate platelets through toll-like-receptor or inflammasome signaling (40). We found that degradation of DNA was very efficient to prevent NET-mediated blood aggregates. This is also in line with previous studies (10, 41). However, to which extent DNA degradation can affect the thrombo-inflammatory properties of histones requires further research. Interestingly, Elaskalani et al. showed that histones did not promote platelet activation and aggregation but other NET-related proteins such as cathepsin G. Taken together, this suggests that the molecular crosstalk between NETs, coagulation and platelets is complex and potentially mediated by various molecules (42). Previously, NETs were considered as a passive scaffold hosting procoagulatory molecules such as histones or tissue factor, which then in turn initiate blood clotting (13). Following our experimental results, we conclude that NETs cannot be considered as a passive component of the clotting process only but as active structures increasing blood viscosity. Moreover, our experiments also indicate that beyond increased blood viscosity the formation of macroscopic clot-like structures and local stasis was dependent on the surface immobilization of NETs at the vascular wall. Blood stasis was previously defined by Virchow as an important trigger of thrombosis (43).

It is known that polymeric macromolecules can affect the flowing properties of liquids (44). To which extent the flow is affected depends on the molecular weight and the composition of the flowing macromolecule. Investigations of DNA solutions indicated a shear thinning, non-Newtonian fluid behavior at low shear rates (< 1 s^-1^). The transition to a more Newtonian fluid at higher shear rates might be connected to an elongation of the DNA molecules into the direction of the flow (20). These findings are also in accordance with the study of Perkins et al. showing that the stretching of single DNA molecules occurred at shear rates in the range of 1 s^-1^ (36). However, properties of polymer solutions under flow are complex and a detailed discussion is beyond the scope of the current manuscript. Here, we aim to emphasize that DNA and in particular NETs are not only scaffolds that harbor procoagulatory molecules but have an intrinsic ability to affect the flow of liquids independent of other plasma proteins or the plasmatic coagulation. Our microparticle velocity experiments indicate that at shear rates above 500 s^-1^ the impact of NETs on fluid viscosity declines. Further *in vivo* experiments using e.g., two-photon microscopy are required to explore the physiological impact of NETs on blood clotting at different shear rates such as low shear rates in large veins (~10 s^-1^) or high shear rates in arterioles and capillaries (~2000 s^-1^) (45, 46).

In comparison to purified DNA, NETs were more potent to affect the flow. This effect is most likely related to a higher structural integrity of NETs as NET-bound proteins such as histones may support DNA chain entanglement by increasing inter- and intramolecular interactions. Although it was previously suggested that NETs can induce the intrinsic coagulation cascade, we measured no activation of factor XII in our experimental setup. However, we found that the complement system was activated during NET formation. Although the trigger of the complement cascade remains to be identified, previous studies suggested the initiation of the alternative pathway by NET exposed molecules (9). Next to NETs, neutrophils produce and secrete the complement activating factor properdin indicating a deeply interwoven crosstalk between neutrophils, NETs and the complement. We have recently shown that the C5a mediated recruitment of neutrophils into melanoma tissue was followed by NETosis (23). Interestingly, *in vitro* studies showed that C5a stimulation of neutrophils resulted in augmented production of reactive oxygen species (ROS) and NETosis (47). In our previous work, we found that NETosis requires the additional formation of the MAC on the surface of neutrophils (23). The MAC is the terminal end point of the complement cascade and a pore forming lipophilic multiprotein complex that permeabilizes the plasma membrane of the attacked cell. A requirement for MAC deposition was the close spatial proximity between the endothelium and the recruited neutrophils enabling the transfer of MAC progenitor complexes from the endothelial to the neutrophil surface. In the present manuscript, we also found that the activation of the complement system at the endothelial surface facilitated NETosis and blood aggregation. In contrast, complement activation in the fluid phase triggered cell-free DNA release but failed to induce the formation of clot-like structures. To completely understand acting molecular players, further research is required. In particular, the role of C5a as a neutrophil attractant molecule and NETosis trigger requires further attention in future research. Next to C5a, the deposition of C3b at the endothelial surface can promote complement receptor-mediated adhesion and activation of neutrophils, which in turn may amplify neutrophil aggregation and NET formation at the vascular wall (48, 49).

In a clinical context, intra-vascular NETs were detected at vasculitic lesions and in thrombi from anti-neutrophil cytoplasmic antibody (ANCA) associated vasculitis (AAV) patients (50). A direct tissue damaging effect of NETs and NET components on the endothelium has also been described and was associated to the activation of the alternative complement pathway (51). Beyond that, elevated markers of NETs or NET-associated proteins were found in AAV patient sera (52). Clinical investigations of AAV patients suggested that insufficient NET degradation due to a reduced DNase I I activity promoted thrombosis (53). Recent clinical trials indicate that inhibition of the C5a receptor 1 by avacopan is beneficial for AAV patients suggesting a relevant involvement of the complement system (54, 55). To our knowledge, whether such inhibition prevents NETosis and thrombo-inflammation has not yet been investigated. In the context of our data, inhibition of C5a signaling might be a potent drug to prevent recruitment and clustering of neutrophils at the endothelium counteracting the pro-thrombotic release of NETs at the vascular wall. To further understand the pathophysiological relevance of MAC for NETosis, it would be highly interesting to study the clinical effect of specific MAC inhibitors.

In conclusion, we showed that vascular surface associated NETs were able to decelerate blood flow and to induce blood stasis even in the absence of other coagulation factors. NET-induced blood aggregation was also tightly connected to the activation of the complement system. Our data suggests that administration of DNase I I or complement inhibiting drugs to patients suffering from NET-mediated thrombotic events might be a straightforward therapeutic approach and probably even more efficient than the exclusive inhibition of the plasmatic coagulation cascade. Pre-clinical data already indicate that DNase I I treatment can counteract thrombosis formation *in vivo* (56, 57). To which extent these data are transferable to a clinical setting and whether NET degradation cause non-indented pro-inflammatory effects due to the release of toxic DNA-bound proteins such as histones requires further research (58). Recent literature reported on a novel therapeutic anti-citrullinated protein antibody (tACPA) inhibiting NET release, improving NET clearance and preventing tissue injury in different NET-mediated pathologies (59).

## Data availability statement

The raw data supporting the conclusions of this article will be made available by the authors, without undue reservation.

## Ethics statement

Ethical review and approval was not required for the study on human participants in accordance with the local legislation and institutional requirements. Written informed consent for participation was not required for this study in accordance with the national legislation and the institutional requirements.

## Author contributions

AB performed most of the research, analyzed the data and wrote the manuscript. SV-Y-S, XL, YW, SK, and TT performed research and interpreted data. CM performed image analysis. KH, VH, and SS supervised experiments and data interpretation. CG designed and conceived the conducted research, interpreted and analyzed data and wrote the manuscript. All authors revised and approved the manuscript.
